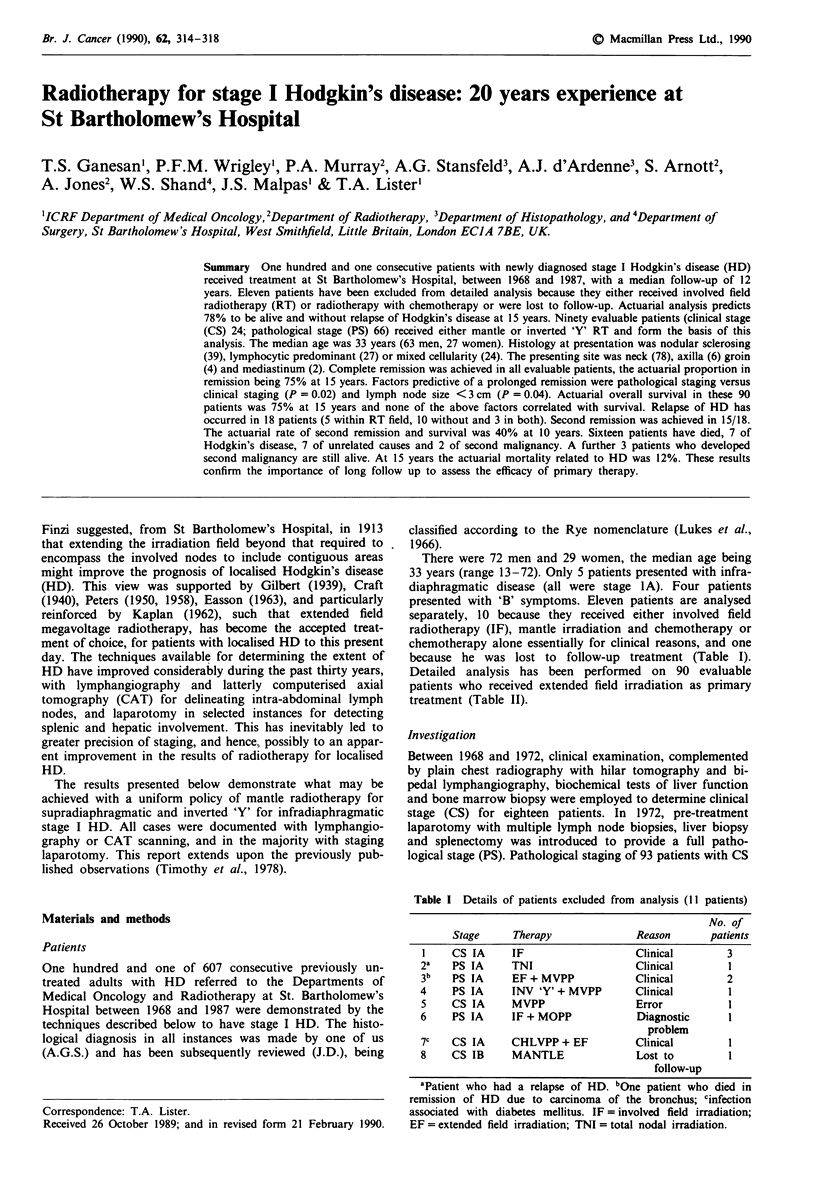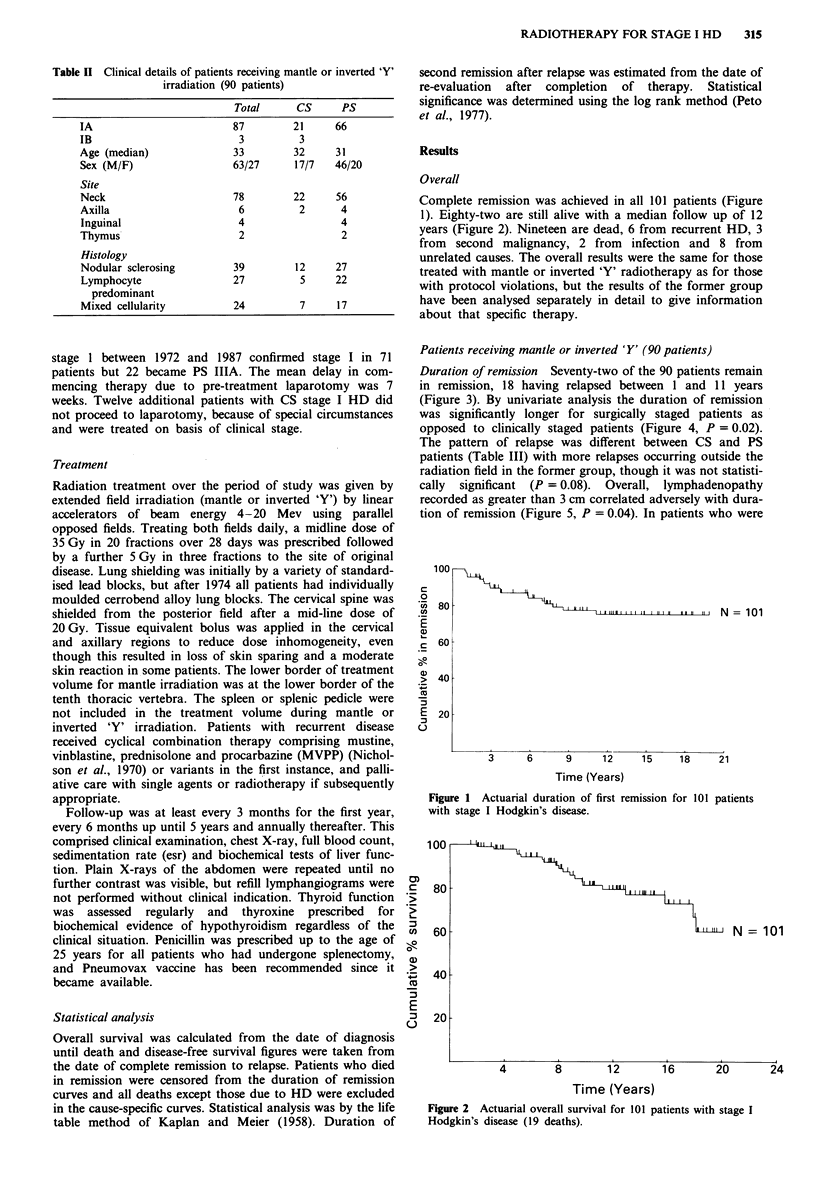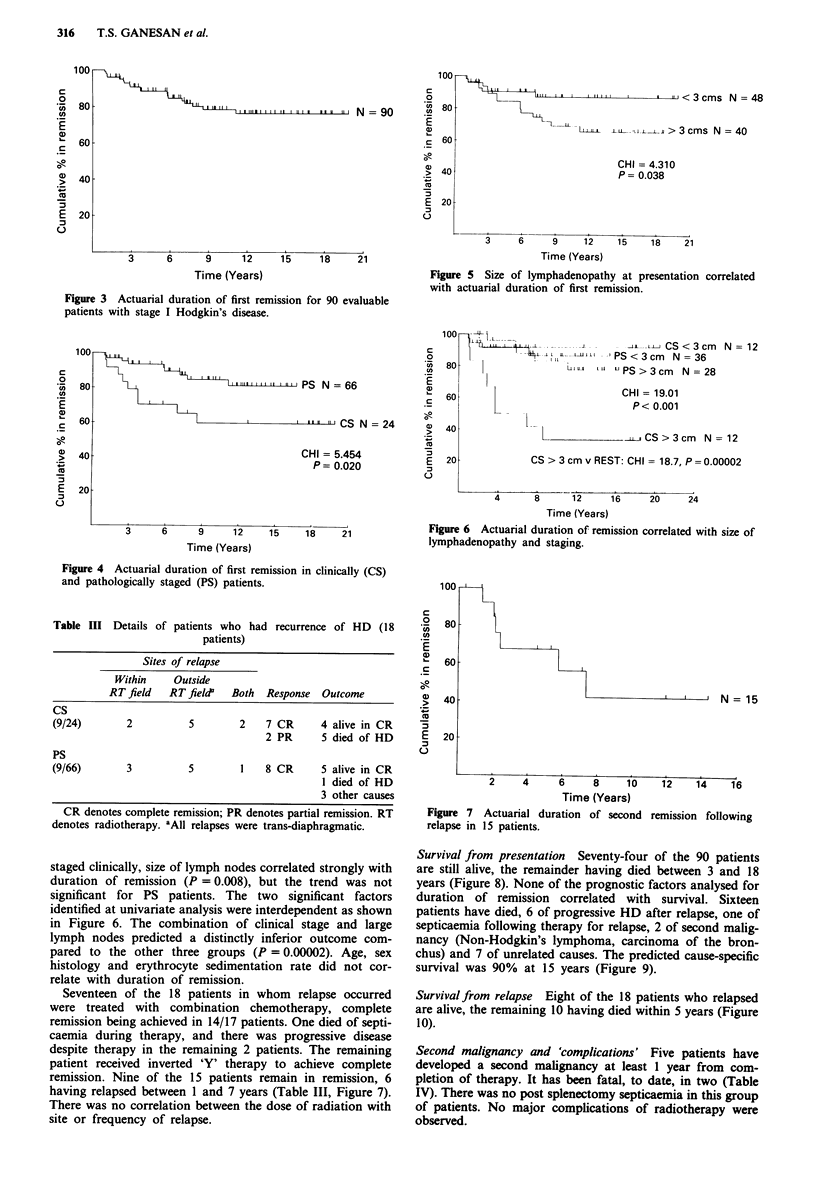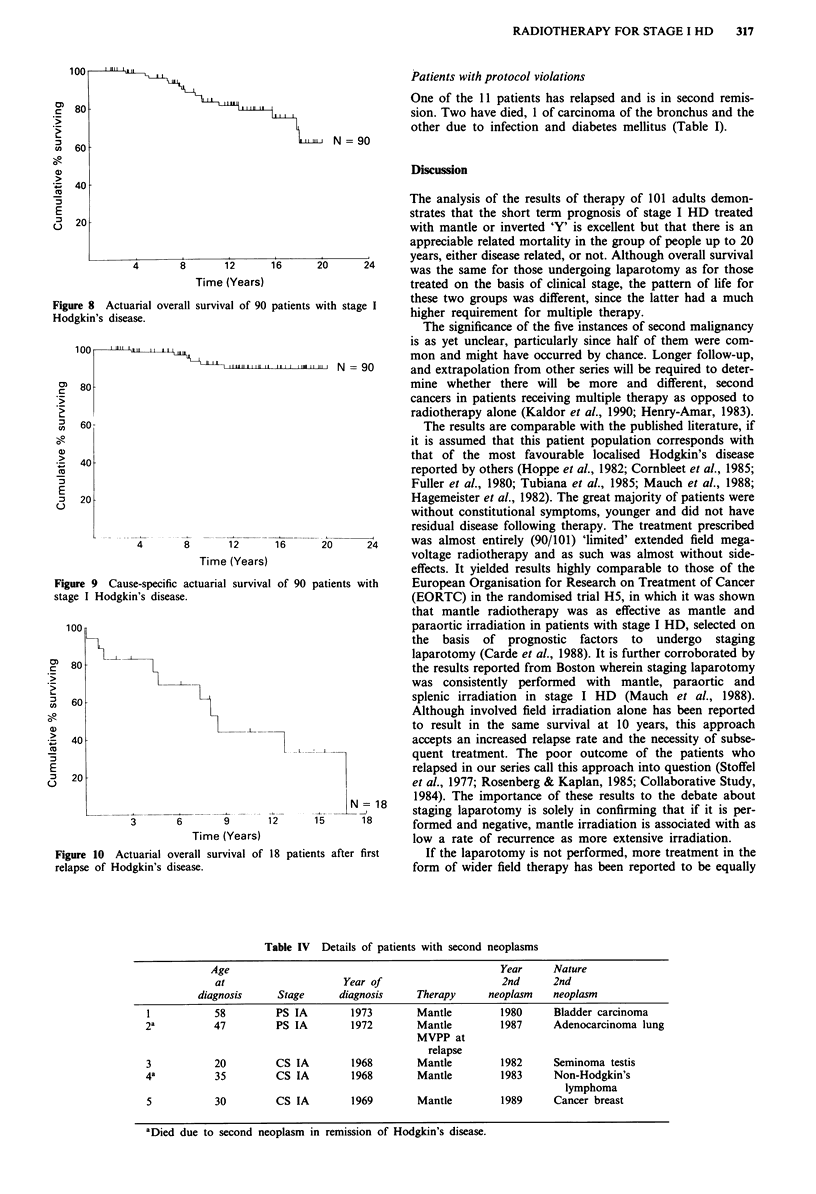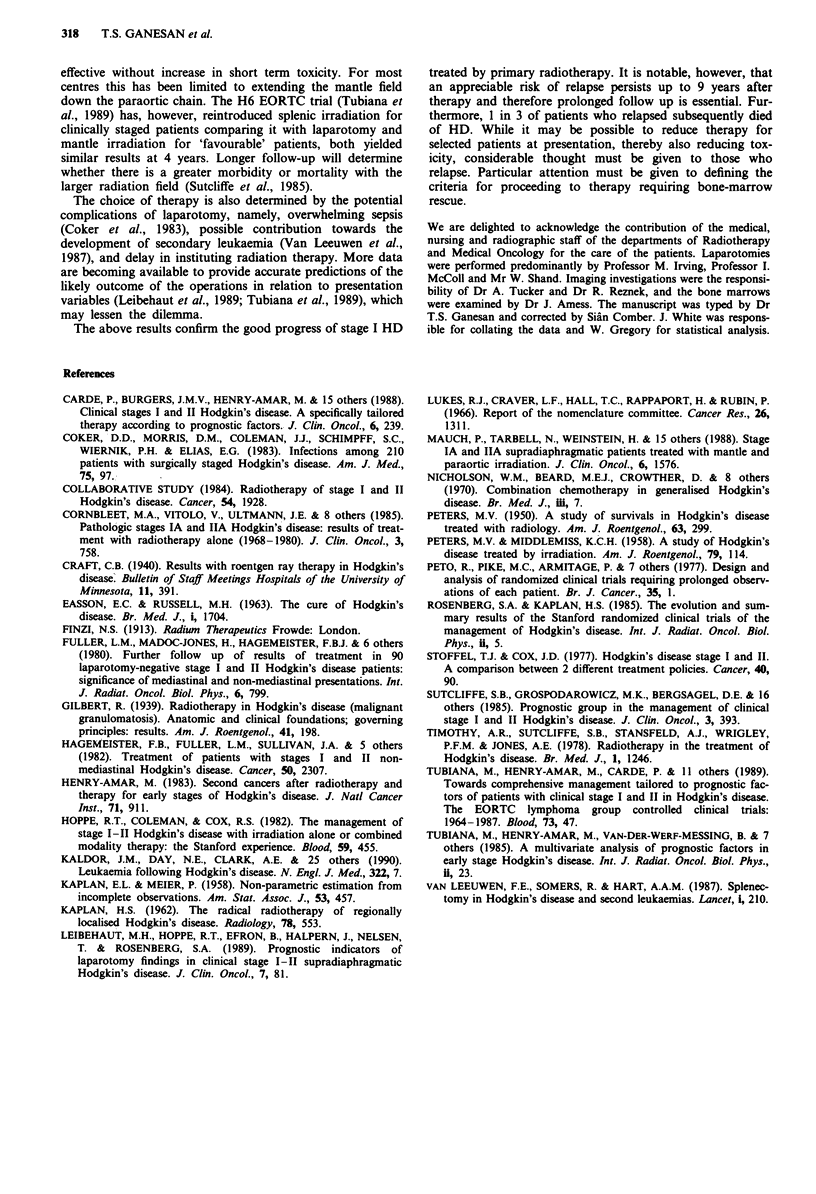# Radiotherapy for stage I Hodgkin's disease: 20 years experience at St Bartholomew's Hospital.

**DOI:** 10.1038/bjc.1990.285

**Published:** 1990-08

**Authors:** T. S. Ganesan, P. F. Wrigley, P. A. Murray, A. G. Stansfeld, A. J. d'Ardenne, S. Arnott, A. Jones, W. S. Shand, J. S. Malpas, T. A. Lister

**Affiliations:** ICRF Department of Medical Oncology, St Bartholomew's Hospital, West Smithfield, Little Britain, London, UK.

## Abstract

One hundred and one consecutive patients with newly diagnosed stage I Hodgkin's disease (HD) received treatment at St Bartholomew's Hospital, between 1968 and 1987, with a median follow-up of 12 years. Eleven patients have been excluded from detailed analysis because they either received involved field radiotherapy (RT) or radiotherapy with chemotherapy or were lost to follow-up. Actuarial analysis predicts 78% to be alive and without relapse of Hodgkin's disease at 15 years. Ninety evaluable patients (clinical stage (CS) 24; pathological stage (PS) 66) received either mantle or inverted 'Y' RT and form the basis of this analysis. The median age was 33 years (63 men, 27 women). Histology at presentation was nodular sclerosing (39), lymphocytic predominant (27) or mixed cellularity (24). The presenting site was neck (78), axilla (6) groin (4) and mediastinum (2). Complete remission was achieved in all evaluable patients, the actuarial proportion in remission being 75% at 15 years. Factors predictive of a prolonged remission were pathological staging versus clinical staging (P = 0.02) and lymph node size less than 3 cm (P = 0.04). Actuarial overall survival in these 90 patients was 75% at 15 years and none of the above factors correlated with survival. Relapse of HD has occurred in 18 patients (5 within RT field, 10 without and 3 in both). Second remission was achieved in 15/18. The actuarial rate of second remission and survival was 40% at 10 years. Sixteen patients have died, 7 of Hodgkin's disease, 7 of unrelated causes and 2 of second malignancy. A further 3 patients who developed second malignancy are still alive. At 15 years the actuarial mortality related to HD was 12%. These results confirm the importance of long follow up to assess the efficacy of primary therapy.


Br. J. Cancer (1990), 62, 314–318 © Macmillan Press Ltd., 1990

### Radiotherapy for stage I Hodgkin's disease: 20 years experience at St Bartholomew's Hospital

T.S. Ganesan[1], P.F.M. Wrigley[1], P.A. Murray[2], A.G. Stansfeld[3], A.J. d'Ardenne[3], S. Arnott[2], A. Jones[2], W.S. Shand[4], J.S. Malpas[1] & T.A. Lister[1]

[1]*ICRF Department of Medical Oncology,* [2]*Department of Radiotherapy,* [3]*Department of Histopathology, and* [4]*Department of Surgery, St Bartholomew's Hospital, West Smithfield, Little Britain, London EC1A 7BE, UK.*

**Summary** One hundred and one consecutive patients with newly diagnosed stage I Hodgkin's disease (HD) received treatment at St Bartholomew's Hospital, between 1968 and 1987, with a median follow-up of 12 years. Eleven patients have been excluded from detailed analysis because they either received involved field radiotherapy (RT) or radiotherapy with chemotherapy or were lost to follow-up. Actuarial analysis predicts 78% to be alive and without relapse of Hodgkin's disease at 15 years. Ninety evaluable patients (clinical stage (CS) 24; pathological stage (PS) 66) received either mantle or inverted 'Y' RT and form the basis of this analysis. The median age was 33 years (63 men, 27 women). Histology at presentation was nodular sclerosing (39), lymphocytic predominant (27) or mixed cellularity (24). The presenting site was neck (78), axilla (6) groin (4) and mediastinum (2). Complete remission was achieved in all evaluable patients, the actuarial proportion in remission being 75% at 15 years. Factors predictive of a prolonged remission were pathological staging versus clinical staging ($P = 0.02$) and lymph node size $<3$ cm ($P = 0.04$). Actuarial overall survival in these 90 patients was 75% at 15 years and none of the above factors correlated with survival. Relapse of HD has occurred in 18 patients (5 within RT field, 10 without and 3 in both). Second remission was achieved in 15/18. The actuarial rate of second remission and survival was 40% at 10 years. Sixteen patients have died, 7 of Hodgkin's disease, 7 of unrelated causes and 2 of second malignancy. A further 3 patients who developed second malignancy are still alive. At 15 years the actuarial mortality related to HD was 12%. These results confirm the importance of long follow up to assess the efficacy of primary therapy.

Finzi suggested, from St Bartholomew's Hospital, in 1913 that extending the irradiation field beyond that required to encompass the involved nodes to include contiguous areas might improve the prognosis of localised Hodgkin's disease (HD). This view was supported by Gilbert (1939), Craft (1940), Peters (1950, 1958), Easson (1963), and particularly reinforced by Kaplan (1962), such that extended field megavoltage radiotherapy, has become the accepted treatment of choice, for patients with localised HD to this present day. The techniques available for determining the extent of HD have improved considerably during the past thirty years, with lymphangiography and latterly computerised axial tomography (CAT) for delineating intra-abdominal lymph nodes, and laparotomy in selected instances for detecting splenic and hepatic involvement. This has inevitably led to greater precision of staging, and hence, possibly to an apparent improvement in the results of radiotherapy for localised HD.

The results presented below demonstrate what may be achieved with a uniform policy of mantle radiotherapy for supradiaphragmatic and inverted 'Y' for infradiaphragmatic stage I HD. All cases were documented with lymphangiography or CAT scanning, and in the majority with staging laparotomy. This report extends upon the previously published observations (Timothy *et al.*, 1978).

### Materials and methods

### *Patients*

One hundred and one of 607 consecutive previously untreated adults with HD referred to the Departments of Medical Oncology and Radiotherapy at St. Bartholomew's Hospital between 1968 and 1987 were demonstrated by the techniques described below to have stage I HD. The histological diagnosis in all instances was made by one of us (A.G.S.) and has been subsequently reviewed (J.D.), being classified according to the Rye nomenclature (Lukes *et al.*, 1966).

There were 72 men and 29 women, the median age being 33 years (range 13–72). Only 5 patients presented with infradiaphragmatic disease (all were stage 1A). Four patients presented with 'B' symptoms. Eleven patients are analysed separately, 10 because they received either involved field radiotherapy (IF), mantle irradiation and chemotherapy or chemotherapy alone essentially for clinical reasons, and one because he was lost to follow-up treatment (Table I). Detailed analysis has been performed on 90 evaluable patients who received extended field irradiation as primary treatment (Table II).

### *Investigation*

Between 1968 and 1972, clinical examination, complemented by plain chest radiography with hilar tomography and bipedal lymphangiography, biochemical tests of liver function and bone marrow biopsy were employed to determine clinical stage (CS) for eighteen patients. In 1972, pre-treatment laparotomy with multiple lymph node biopsies, liver biopsy and splenectomy was introduced to provide a full pathological stage (PS). Pathological staging of 93 patients with CS

**Table I** Details of patients excluded from analysis (11 patients)

| | *Stage* | *Therapy* | *Reason* | *No. of patients* |
|---|---|---|---|---|
| 1 | CS IA | IF | Clinical | 3 |
| 2[a] | PS IA | TNI | Clinical | 1 |
| 3[b] | PS IA | EF + MVPP | Clinical | 2 |
| 4 | PS IA | INV 'Y' + MVPP | Clinical | 1 |
| 5 | CS IA | MVPP | Error | 1 |
| 6 | PS IA | IF + MOPP | Diagnostic problem | 1 |
| 7[c] | CS IA | CHLVPP + EF | Clinical | 1 |
| 8 | CS IB | MANTLE | Lost to follow-up | 1 |

[a]Patient who had a relapse of HD. [b]One patient who died in remission of HD due to carcinoma of the bronchus; [c]infection associated with diabetes mellitus. IF = involved field irradiation; EF = extended field irradiation; TNI = total nodal irradiation.

Correspondence: T.A. Lister.
Received 26 October 1989; and in revised form 21 February 1990.

**Table II** Clinical details of patients receiving mantle or inverted 'Y' irradiation (90 patients)

| | *Total* | *CS* | *PS* |
|---|---|---|---|
| IA | 87 | 21 | 66 |
| IB | 3 | 3 | |
| Age (median) | 33 | 32 | 31 |
| Sex (M/F) | 63/27 | 17/7 | 46/20 |
| *Site* | | | |
| Neck | 78 | 22 | 56 |
| Axilla | 6 | 2 | 4 |
| Inguinal | 4 | | 4 |
| Thymus | 2 | | 2 |
| *Histology* | | | |
| Nodular sclerosing | 39 | 12 | 27 |
| Lymphocyte predominant | 27 | 5 | 22 |
| Mixed cellularity | 24 | 7 | 17 |

stage 1 between 1972 and 1987 confirmed stage I in 71 patients but 22 became PS IIIA. The mean delay in commencing therapy due to pre-treatment laparotomy was 7 weeks. Twelve additional patients with CS stage I HD did not proceed to laparotomy, because of special circumstances and were treated on basis of clinical stage.

### *Treatment*

Radiation treatment over the period of study was given by extended field irradiation (mantle or inverted 'Y') by linear accelerators of beam energy 4–20 Mev using parallel opposed fields. Treating both fields daily, a midline dose of 35 Gy in 20 fractions over 28 days was prescribed followed by a further 5 Gy in three fractions to the site of original disease. Lung shielding was initially by a variety of standardised lead blocks, but after 1974 all patients had individually moulded cerrobend alloy lung blocks. The cervical spine was shielded from the posterior field after a mid-line dose of 20 Gy. Tissue equivalent bolus was applied in the cervical and axillary regions to reduce dose inhomogeneity, even though this resulted in loss of skin sparing and a moderate skin reaction in some patients. The lower border of treatment volume for mantle irradiation was at the lower border of the tenth thoracic vertebra. The spleen or splenic pedicle were not included in the treatment volume during mantle or inverted 'Y' irradiation. Patients with recurrent disease received cyclical combination therapy comprising mustine, vinblastine, prednisolone and procarbazine (MVPP) (Nicholson *et al.*, 1970) or variants in the first instance, and palliative care with single agents or radiotherapy if subsequently appropriate.

Follow-up was at least every 3 months for the first year, every 6 months up until 5 years and annually thereafter. This comprised clinical examination, chest X-ray, full blood count, sedimentation rate (esr) and biochemical tests of liver function. Plain X-rays of the abdomen were repeated until no further contrast was visible, but refill lymphangiograms were not performed without clinical indication. Thyroid function was assessed regularly and thyroxine prescribed for biochemical evidence of hypothyroidism regardless of the clinical situation. Penicillin was prescribed up to the age of 25 years for all patients who had undergone splenectomy, and Pneumovax vaccine has been recommended since it became available.

### *Statistical analysis*

Overall survival was calculated from the date of diagnosis until death and disease-free survival figures were taken from the date of complete remission to relapse. Patients who died in remission were censored from the duration of remission curves and all deaths except those due to HD were excluded in the cause-specific curves. Statistical analysis was by the life table method of Kaplan and Meier (1958). Duration of second remission after relapse was estimated from the date of re-evaluation after completion of therapy. Statistical significance was determined using the log rank method (Peto *et al.*, 1977).

### Results

### *Overall*

Complete remission was achieved in all 101 patients (Figure 1). Eighty-two are still alive with a median follow up of 12 years (Figure 2). Nineteen are dead, 6 from recurrent HD, 3 from second malignancy, 2 from infection and 8 from unrelated causes. The overall results were the same for those treated with mantle or inverted 'Y' radiotherapy as for those with protocol violations, but the results of the former group have been analysed separately in detail to give information about that specific therapy.

### *Patients receiving mantle or inverted 'Y' (90 patients)*

*Duration of remission* Seventy-two of the 90 patients remain in remission, 18 having relapsed between 1 and 11 years (Figure 3). By univariate analysis the duration of remission was significantly longer for surgically staged patients as opposed to clinically staged patients (Figure 4, $P = 0.02$). The pattern of relapse was different between CS and PS patients (Table III) with more relapses occurring outside the radiation field in the former group, though it was not statistically significant ($P = 0.08$). Overall, lymphadenopathy recorded as greater than 3 cm correlated adversely with duration of remission (Figure 5, $P = 0.04$). In patients who were

**Figure 1** Actuarial duration of first remission for 101 patients with stage I Hodgkin's disease.

**Figure 2** Actuarial overall survival for 101 patients with stage I Hodgkin's disease (19 deaths).

**Figure 3** Actuarial duration of first remission for 90 evaluable patients with stage I Hodgkin's disease.

**Figure 4** Actuarial duration of first remission in clinically (CS) and pathologically staged (PS) patients.

**Table III** Details of patients who had recurrence of HD (18 patients)

| | *Sites of relapse* | | | | |
|---|---|---|---|---|---|
| | *Within RT field* | *Outside RT field*[a] | *Both* | *Response* | *Outcome* |
| CS (9/24) | 2 | 5 | 2 | 7 CR<br>2 PR | 4 alive in CR<br>5 died of HD |
| PS (9/66) | 3 | 5 | 1 | 8 CR | 5 alive in CR<br>1 died of HD<br>3 other causes |

CR denotes complete remission; PR denotes partial remission. RT denotes radiotherapy. [a]All relapses were trans-diaphragmatic.

staged clinically, size of lymph nodes correlated strongly with duration of remission ($P = 0.008$), but the trend was not significant for PS patients. The two significant factors identified at univariate analysis were interdependent as shown in Figure 6. The combination of clinical stage and large lymph nodes predicted a distinctly inferior outcome compared to the other three groups ($P = 0.00002$). Age, sex histology and erythrocyte sedimentation rate did not correlate with duration of remission.

Seventeen of the 18 patients in whom relapse occurred were treated with combination chemotherapy, complete remission being achieved in 14/17 patients. One died of septicaemia during therapy, and there was progressive disease despite therapy in the remaining 2 patients. The remaining patient received inverted 'Y' therapy to achieve complete remission. Nine of the 15 patients remain in remission, 6 having relapsed between 1 and 7 years (Table III, Figure 7). There was no correlation between the dose of radiation with site or frequency of relapse.

**Figure 5** Size of lymphadenopathy at presentation correlated with actuarial duration of first remission.

**Figure 6** Actuarial duration of remission correlated with size of lymphadenopathy and staging.

**Figure 7** Actuarial duration of second remission following relapse in 15 patients.

*Survival from presentation* Seventy-four of the 90 patients are still alive, the remainder having died between 3 and 18 years (Figure 8). None of the prognostic factors analysed for duration of remission correlated with survival. Sixteen patients have died, 6 of progressive HD after relapse, one of septicaemia following therapy for relapse, 2 of second malignancy (Non-Hodgkin's lymphoma, carcinoma of the bronchus) and 7 of unrelated causes. The predicted cause-specific survival was 90% at 15 years (Figure 9).

*Survival from relapse* Eight of the 18 patients who relapsed are alive, the remaining 10 having died within 5 years (Figure 10).

*Second malignancy and 'complications'* Five patients have developed a second malignancy at least 1 year from completion of therapy. It has been fatal, to date, in two (Table IV). There was no post splenectomy septicaemia in this group of patients. No major complications of radiotherapy were observed.

**Figure 8** Actuarial overall survival of 90 patients with stage I Hodgkin's disease.

**Figure 9** Cause-specific actuarial survival of 90 patients with stage I Hodgkin's disease.

**Figure 10** Actuarial overall survival of 18 patients after first relapse of Hodgkin's disease.

### *Patients with protocol violations*

One of the 11 patients has relapsed and is in second remission. Two have died, 1 of carcinoma of the bronchus and the other due to infection and diabetes mellitus (Table I).

### Discussion

The analysis of the results of therapy of 101 adults demonstrates that the short term prognosis of stage I HD treated with mantle or inverted 'Y' is excellent but that there is an appreciable related mortality in the group of people up to 20 years, either disease related, or not. Although overall survival was the same for those undergoing laparotomy as for those treated on the basis of clinical stage, the pattern of life for these two groups was different, since the latter had a much higher requirement for multiple therapy.

The significance of the five instances of second malignancy is as yet unclear, particularly since half of them were common and might have occurred by chance. Longer follow-up, and extrapolation from other series will be required to determine whether there will be more and different, second cancers in patients receiving multiple therapy as opposed to radiotherapy alone (Kaldor *et al.*, 1990; Henry-Amar, 1983).

The results are comparable with the published literature, if it is assumed that this patient population corresponds with that of the most favourable localised Hodgkin's disease reported by others (Hoppe *et al.*, 1982; Cornbleet *et al.*, 1985; Fuller *et al.*, 1980; Tubiana *et al.*, 1985; Mauch *et al.*, 1988; Hagemeister *et al.*, 1982). The great majority of patients were without constitutional symptoms, younger and did not have residual disease following therapy. The treatment prescribed was almost entirely (90/101) 'limited' extended field megavoltage radiotherapy and as such was almost without side-effects. It yielded results highly comparable to those of the European Organisation for Research on Treatment of Cancer (EORTC) in the randomised trial H5, in which it was shown that mantle radiotherapy was as effective as mantle and paraortic irradiation in patients with stage I HD, selected on the basis of prognostic factors to undergo staging laparotomy (Carde *et al.*, 1988). It is further corroborated by the results reported from Boston wherein staging laparotomy was consistently performed with mantle, paraortic and splenic irradiation in stage I HD (Mauch *et al.*, 1988). Although involved field irradiation alone has been reported to result in the same survival at 10 years, this approach accepts an increased relapse rate and the necessity of subsequent treatment. The poor outcome of the patients who relapsed in our series call this approach into question (Stoffel *et al.*, 1977; Rosenberg & Kaplan, 1985; Collaborative Study, 1984). The importance of these results to the debate about staging laparotomy is solely in confirming that if it is performed and negative, mantle irradiation is associated with as low a rate of recurrence as more extensive irradiation.

If the laparotomy is not performed, more treatment in the form of wider field therapy has been reported to be equally

**Table IV** Details of patients with second neoplasms

| | *Age at diagnosis* | *Stage* | *Year of diagnosis* | *Therapy* | *Year 2nd neoplasm* | *Nature 2nd neoplasm* |
|---|---|---|---|---|---|---|
| 1 | 58 | PS IA | 1973 | Mantle | 1980 | Bladder carcinoma |
| 2[a] | 47 | PS IA | 1972 | Mantle MVPP at relapse | 1987 | Adenocarcinoma lung |
| 3 | 20 | CS IA | 1968 | Mantle | 1982 | Seminoma testis |
| 4[a] | 35 | CS IA | 1968 | Mantle | 1983 | Non-Hodgkin's lymphoma |
| 5 | 30 | CS IA | 1969 | Mantle | 1989 | Cancer breast |

[a]Died due to second neoplasm in remission of Hodgkin's disease.

effective without increase in short term toxicity. For most centres this has been limited to extending the mantle field down the paraortic chain. The H6 EORTC trial (Tubiana *et al.*, 1989) has, however, reintroduced splenic irradiation for clinically staged patients comparing it with laparotomy and mantle irradiation for 'favourable' patients, both yielded similar results at 4 years. Longer follow-up will determine whether there is a greater morbidity or mortality with the larger radiation field (Sutcliffe *et al.*, 1985).

The choice of therapy is also determined by the potential complications of laparotomy, namely, overwhelming sepsis (Coker *et al.*, 1983), possible contribution towards the development of secondary leukaemia (Van Leeuwen *et al.*, 1987), and delay in instituting radiation therapy. More data are becoming available to provide accurate predictions of the likely outcome of the operations in relation to presentation variables (Leibehaut *et al.*, 1989; Tubiana *et al.*, 1989), which may lessen the dilemma.

The above results confirm the good progress of stage I HD treated by primary radiotherapy. It is notable, however, that an appreciable risk of relapse persists up to 9 years after therapy and therefore prolonged follow up is essential. Furthermore, 1 in 3 of patients who relapsed subsequently died of HD. While it may be possible to reduce therapy for selected patients at presentation, thereby also reducing toxicity, considerable thought must be given to those who relapse. Particular attention must be given to defining the criteria for proceeding to therapy requiring bone-marrow rescue.

We are delighted to acknowledge the contribution of the medical, nursing and radiographic staff of the departments of Radiotherapy and Medical Oncology for the care of the patients. Laparotomies were performed predominantly by Professor M. Irving, Professor I. McColl and Mr W. Shand. Imaging investigations were the responsibility of Dr A. Tucker and Dr R. Reznek, and the bone marrows were examined by Dr J. Amess. The manuscript was typed by Dr T.S. Ganesan and corrected by Siân Comber. J. White was responsible for collating the data and W. Gregory for statistical analysis.

### References

CARDE, P., BURGERS, J.M.V., HENRY-AMAR, M. & 15 others (1988). Clinical stages I and II Hodgkin's disease. A specifically tailored therapy according to prognostic factors. *J. Clin. Oncol.*, **6**, 239.

COKER, D.D., MORRIS, D.M., COLEMAN, J.J., SCHIMPFF, S.C., WIERNIK, P.H. & ELIAS, E.G. (1983). Infections among 210 patients with surgically staged Hodgkin's disease. *Am. J. Med.*, **75**, 97.

COLLABORATIVE STUDY (1984). Radiotherapy of stage I and II Hodgkin's disease. *Cancer*, **54**, 1928.

CORNBLEET, M.A., VITOLO, V., ULTMANN, J.E. & 8 others (1985). Pathologic stages IA and IIA Hodgkin's disease: results of treatment with radiotherapy alone (1968–1980). *J. Clin. Oncol.*, **3**, 758.

CRAFT, C.B. (1940). Results with roentgen ray therapy in Hodgkin's disease. *Bulletin of Staff Meetings Hospitals of the University of Minnesota*, **11**, 391.

EASSON, E.C. & RUSSELL, M.H. (1963). The cure of Hodgkin's disease. *Br. Med. J.*, **i**, 1704.

FINZI, N.S. (1913). *Radium Therapeutics* Frowde: London.

FULLER, L.M., MADOC-JONES, H., HAGEMEISTER, F.B.J. & 6 others (1980). Further follow up of results of treatment in 90 laparotomy-negative stage I and II Hodgkin's disease patients: significance of mediastinal and non-mediastinal presentations. *Int. J. Radiat. Oncol. Biol. Phys.*, **6**, 799.

GILBERT, R. (1939). Radiotherapy in Hodgkin's disease (malignant granulomatosis). Anatomic and clinical foundations; governing principles: results. *Am. J. Roentgenol.*, **41**, 198.

HAGEMEISTER, F.B., FULLER, L.M., SULLIVAN, J.A. & 5 others (1982). Treatment of patients with stages I and II non-mediastinal Hodgkin's disease. *Cancer*, **50**, 2307.

HENRY-AMAR, M. (1983). Second cancers after radiotherapy and therapy for early stages of Hodgkin's disease. *J. Natl Cancer Inst.*, **71**, 911.

HOPPE, R.T., COLEMAN, & COX, R.S. (1982). The management of stage I–II Hodgkin's disease with irradiation alone or combined modality therapy: the Stanford experience. *Blood*, **59**, 455.

KALDOR, J.M., DAY, N.E., CLARK, A.E. & 25 others (1990). Leukaemia following Hodgkin's disease. *N. Engl. J. Med.*, **322**, 7.

KAPLAN, E.L. & MEIER, P. (1958). Non-parametric estimation from incomplete observations. *Am. Stat. Assoc. J.*, **53**, 457.

KAPLAN, H.S. (1962). The radical radiotherapy of regionally localised Hodgkin's disease. *Radiology*, **78**, 553.

LEIBEHAUT, M.H., HOPPE, R.T., EFRON, B., HALPERN, J., NELSEN, T. & ROSENBERG, S.A. (1989). Prognostic indicators of laparotomy findings in clinical stage I–II supradiaphragmatic Hodgkin's disease. *J. Clin. Oncol.*, **7**, 81.

LUKES, R.J., CRAVER, L.F., HALL, T.C., RAPPAPORT, H. & RUBIN, P. (1966). Report of the nomenclature committee. *Cancer Res.*, **26**, 1311.

MAUCH, P., TARBELL, N., WEINSTEIN, H. & 15 others (1988). Stage IA and IIA supradiaphragmatic patients treated with mantle and paraortic irradiation. *J. Clin. Oncol.*, **6**, 1576.

NICHOLSON, W.M., BEARD, M.E.J., CROWTHER, D. & 8 others (1970). Combination chemotherapy in generalised Hodgkin's disease. *Br. Med. J.*, **iii**, 7.

PETERS, M.V. (1950). A study of survivals in Hodgkin's disease treated with radiology. *Am. J. Roentgenol.*, **63**, 299.

PETERS, M.V. & MIDDLEMISS, K.C.H. (1958). A study of Hodgkin's disease treated by irradiation. *Am. J. Roentgenol.*, **79**, 114.

PETO, R., PIKE, M.C., ARMITAGE, P. & 7 others (1977). Design and analysis of randomized clinical trials requiring prolonged observations of each patient. *Br. J. Cancer.*, **35**, 1.

ROSENBERG, S.A. & KAPLAN, H.S. (1985). The evolution and summary results of the Stanford randomized clinical trials of the management of Hodgkin's disease. *Int. J. Radiat. Oncol. Biol. Phys.*, **ii**, 5.

STOFFEL, T.J. & COX, J.D. (1977). Hodgkin's disease stage I and II. A comparison between 2 different treatment policies. *Cancer*, **40**, 90.

SUTCLIFFE, S.B., GROSPODAROWICZ, M.K., BERGSAGEL, D.E. & 16 others (1985). Prognostic group in the management of clinical stage I and II Hodgkin's disease. *J. Clin. Oncol.*, **3**, 393.

TIMOTHY, A.R., SUTCLIFFE, S.B., STANSFELD, A.J., WRIGLEY, P.F.M. & JONES, A.E. (1978). Radiotherapy in the treatment of Hodgkin's disease. *Br. Med. J.*, **1**, 1246.

TUBIANA, M., HENRY-AMAR, M., CARDE, P. & 11 others (1989). Towards comprehensive management tailored to prognostic factors of patients with clinical stage I and II in Hodgkin's disease. The EORTC lymphoma group controlled clinical trials: 1964–1987. *Blood*, **73**, 47.

TUBIANA, M., HENRY-AMAR, M., VAN-DER-WERF-MESSING, B. & 7 others (1985). A multivariate analysis of prognostic factors in early stage Hodgkin's disease. *Int. J. Radiat. Oncol. Biol. Phys.*, **ii**, 23.

VAN LEEUWEN, F.E., SOMERS, R. & HART, A.A.M. (1987). Splenectomy in Hodgkin's disease and second leukaemias. *Lancet*, **i**, 210.